# Supplementary material for: Resolving the full spectrum of human genome variation using Linked-Reads
Source: Genome Res. 2019 Apr;29(4):635–45. doi: 10.1101/gr.234443.118 (PMC6442396; doi:10.1101/gr.234443.118)
Supplement: Supplemental Material [file supp_gr.234443.118_Supplemental_longranger-2.2.zip › longranger-2.2.tar.bz2!/lr2.2-public/lib/go/src/code.google.com/vitess/docs/user-guide/backup-and-restore.html]

Vitess / Backing Up Data


Toggle navigation

- Overview
- Guides
- Reference
- Blog
- GitHub

- #### Overview

  - What is Vitess
  - Scaling MySQL with Vitess
  - Key Concepts
- #### Getting Started

  - Run Vitess on Kubernetes
    - Custom Docker Build
  - Run Vitess Locally
- #### User Guide

  - Introduction- Client Libraries- Backing Up Data- Reparenting
        - Schema Management
        - VSchema Guide
        - Schema Swap (Tutorial)
        - Sharding
          - Horizontal Sharding (Tutorial, automated)
          - Horizontal Sharding (Tutorial, manual)
          - Sharding in Kubernetes (Tutorial, automated)
          - Sharding in Kubernetes (Tutorial, manual)
        - Vitess Sequences
        - MySQL Server Protocol
        - Vitess and Replication
        - Update Stream
        - Row Based Replication
        - Topology Service
        - Transport Security Model
        - Launching
          - Scalability Philosophy
          - Production Planning
          - Server Configuration
          - 2PC Guide
          - Troubleshooting
        - Upgrading
- #### Reference Guides

  - Vitess API- vtctl Commands
- #### Other Resources

  - Presentations- Blog- Roadmap
- #### Contributing

  - Contributing to Vitess- GitHub Workflow- Code Reviews
- GitHub

# Backing Up Data

- #### Overview

  - What is Vitess
  - Scaling MySQL with Vitess
  - Key Concepts
- #### Getting Started

  - Run Vitess on Kubernetes
    - Custom Docker Build
  - Run Vitess Locally
- #### User Guide

  - Introduction- Client Libraries- Backing Up Data- Reparenting
        - Schema Management
        - VSchema Guide
        - Schema Swap (Tutorial)
        - Sharding
          - Horizontal Sharding (Tutorial, automated)
          - Horizontal Sharding (Tutorial, manual)
          - Sharding in Kubernetes (Tutorial, automated)
          - Sharding in Kubernetes (Tutorial, manual)
        - Vitess Sequences
        - MySQL Server Protocol
        - Vitess and Replication
        - Update Stream
        - Row Based Replication
        - Topology Service
        - Transport Security Model
        - Launching
          - Scalability Philosophy
          - Production Planning
          - Server Configuration
          - 2PC Guide
          - Troubleshooting
        - Upgrading
- #### Reference Guides

  - Vitess API- vtctl Commands
- #### Other Resources

  - Presentations- Blog- Roadmap
- #### Contributing

  - Contributing to Vitess- GitHub Workflow- Code Reviews

This document explains how to create and restore data backups with
Vitess. Vitess uses backups for two purposes:

- Provide a point-in-time backup of the data on a tablet.
- Bootstrap new tablets in an existing shard.

## Prerequisites

Vitess stores data backups on a Backup Storage service, which is
a
pluggable interface.

Currently, we have plugins for:

- A network-mounted path (e.g. NFS)
- Google Cloud Storage
- Amazon S3
- Ceph

Before you can back up or restore a tablet, you need to ensure that the
tablet is aware of the Backup Storage system that you are using. To do so,
use the following command-line flags when starting a vttablet that has
access to the location where you are storing backups.

| Flags | |
| --- | --- |
| `backup_storage_implementation` | Specifies the implementation of the Backup Storage interface to use.   Current plugin options available are:  - `file`: NFS or any other filesystem-mounted network   drive. - `gcs`: Google Cloud Storage. - `s3`: Amazon S3. - `ceph`: Ceph Object Gateway S3 API. |
| `backup_storage_hook` | If set, the contents of every file to backup is sent to a hook. The hook receives the data for each file on stdin. It should echo the transformed data to stdout. Anything the hook prints to stderr will be printed in the vttablet logs.  Hooks should be located in the `vthook` subdirectory of the `VTROOT` directory.  The hook receives a `-operation write` or a `-operation read` parameter depending on the direction of the data processing. For instance, `write` would be for encryption, and `read` would be for decryption. |
| `backup_storage_compress` | This flag controls if the backups are compressed by the Vitess code. By default it is set to true. Use `-backup_storage_compress=false` to disable. This is meant to be used with a `-backup_storage_hook` hook that already compresses the data, to avoid compressing the data twice. |
| `file_backup_storage_root` | For the `file` plugin, this identifies the root directory for backups. |
| `gcs_backup_storage_bucket` | For the `gcs` plugin, this identifies the bucket to use. |
| `s3_backup_aws_region` | For the `s3` plugin, this identifies the AWS region. |
| `s3_backup_storage_bucket` | For the `s3` plugin, this identifies the AWS S3 bucket. |
| `ceph_backup_storage_config` | For the `ceph` plugin, this identifies the path to a text file with a JSON object as configuration. The JSON object requires the following keys: `accessKey`, `secretKey`, `endPoint` and `useSSL`. Bucket name is computed from keyspace name and shard name and is separate for different keyspaces / shards. |
| `restore_from_backup` | Indicates that, when started with an empty MySQL instance, the tablet should restore the most recent backup from the specified storage plugin. |

### Authentication

Note that for the Google Cloud Storage plugin, we currently only
support
Application Default Credentials.
It means that access to Cloud Storage is automatically granted by virtue of
the fact that you're already running within Google Compute Engine or Container
Engine.

For this to work, the GCE instances must have been created with
the scope that
grants read-write access to Cloud Storage. When using Container Engine, you can
do this for all the instances it creates by adding `--scopes storage-rw` to the
`gcloud container clusters create` command as shown in the Vitess on Kubernetes
guide.

## Creating a backup

Run the following vtctl command to create a backup:

```
vtctl Backup <tablet-alias>
```

In response to this command, the designated tablet performs the following
sequence of actions:

1. Switches its type to `BACKUP`. After this step, the tablet is no
   longer used by vtgate to serve any query.
2. Stops replication, get the current replication position (to be saved in the
   backup along with the data).
3. Shuts down its mysqld process.
4. Copies the necessary files to the Backup Storage implementation that was
   specified when the tablet was started. Note if this fails, we still keep
   going, so the tablet is not left in an unstable state because of a storage
   failure.
5. Restarts mysqld.
6. Restarts replication (with the right semi-sync flags corresponding to its
   original type, if applicable).
7. Switches its type back to its original type. After this, it will most likely
   be behind on replication, and not used by vtgate for serving until it catches
   up.

## Restoring a backup

When a tablet starts, Vitess checks the value of the
`-restore_from_backup` command-line flag to determine whether
to restore a backup to that tablet.

- If the flag is present, Vitess tries to restore the most recent backup from
  the Backup Storage system when starting the tablet.
- If the flag is absent, Vitess does not try to restore a backup to the
  tablet. This is the equivalent of starting a new tablet in a new shard.

As noted in the Prerequisites section, the flag is
generally enabled all of the time for all of the tablets in a shard.
If Vitess cannot find a backup in the Backup Storage system, it just
starts the vttablet as a new tablet.

```
vttablet ... -backup_storage_implementation=file \
             -file_backup_storage_root=/nfs/XXX \
             -restore_from_backup
```

## Managing backups

**vtctl** provides two commands for managing backups:

- ListBackups displays the
  existing backups for a keyspace/shard in chronological order.

  ```
  vtctl ListBackups <keyspace/shard>
  ```
- RemoveBackup deletes a
  specified backup for a keyspace/shard.

  ```
  RemoveBackup <keyspace/shard> <backup name>
  ```

## Bootstrapping a new tablet

Bootstrapping a new tablet is almost identical to restoring an existing tablet.
The only thing you need to be cautious about is that the tablet specifies its
keyspace, shard and tablet type when it registers itself at the topology.
Specifically, make sure that the following vttablet parameters are set:

```
    -init_keyspace <keyspace>
    -init_shard <shard>
    -init_tablet_type replica|rdonly
```

The bootstrapped tablet will restore the data from the backup and then apply
changes, which occurred after the backup, by restarting replication.

## Backup Frequency

We recommend to take backups regularly e.g. you should set up a cron
job for it.

To determine the proper frequency for creating backups, consider
the amount of time that you keep replication logs and allow enough
time to investigate and fix problems in the event that a backup
operation fails.

For example, suppose you typically keep four days of replication logs
and you create daily backups. In that case, even if a backup fails,
you have at least a couple of days from the time of the failure to
investigate and fix the problem.

## Concurrency

The back-up and restore processes simultaneously copy and either
compress or decompress multiple files to increase throughput. You
can control the concurrency using command-line flags:

- The vtctl Backup command uses the
  `-concurrency` flag.
- vttablet uses the `-restore_concurrency` flag.

If the network link is fast enough, the concurrency matches the CPU
usage of the process during the backup or restore process.

Contact: vitess@googlegroups.com  **·**  
Announcements  **·**  
© 2017 Vitess powered by Google Inc
